# A sustainable green-approach for biofabrication of chitosan nanoparticles, optimization, characterization, its antifungal activity against phytopathogenic *Fusarium culmorum* and antitumor activity

**DOI:** 10.1038/s41598-024-59702-3

**Published:** 2024-05-17

**Authors:** Noura El-Ahmady El-Naggar, Alaa M. Shiha, Hoda Mahrous, A. B. Abeer Mohammed

**Affiliations:** 1 Department of Bioprocess Development, Genetic Engineering and Biotechnology Research Institute, City of Scientific Research and Technological Applications (SRTA-City), New Borg El-Arab City, 21934 Alexandria Egypt; 2 Microbial Biotechnology Department, Genetic Engineering and Biotechnology Research Institute, University of Sadat City, Sadat City, Egypt; 3 Industrial Biotechnology Department, Genetic Engineering and Biotechnology Research Institute, University of Sadat City, Sadat City, Egypt

**Keywords:** Chitosan nanoparticles, Biofabrication, Optimization, Characterization, Antifungal activities, Anticancer activity, Nanoparticles, Biopolymers, Nanoparticles

## Abstract

Chitosan is a natural non-toxic, biocompatible, biodegradable, and mucoadhesive polymer. It also has a broad spectrum of applications such as agriculture, medical fields, cosmetics and food industries. In this investigation, chitosan nanoparticles were produced by an aqueous extract of *Cympopogon citratus* leaves as a reducing agent. According to the SEM and TEM micrographs, CNPs had a spherical shape, and size ranging from 8.08 to 12.01 nm. CNPs have a positively charged surface with a Zeta potential of + 26 mV. The crystalline feature of CNPs is determined by X-ray diffraction. There are many functional groups, including C꞊C, CH_2_-OH, C–O, C-S, N–H, CN, CH and OH were detected by FTIR analysis. As shown by the thermogravimetric study, CNPs have a high thermal stability. For the optimization of the green synthesis of CNPs, a Face centered central composite design (FCCCD) with 30 trials was used. The maximum yield of CNPs (13.99 mg CNPs/mL) was produced with chitosan concentration 1.5%, pH 4.5 at 40 °C, and incubation period of 30 min. The antifungal activity of CNPs was evaluated against phytopathogenic fungus; *Fusarium culmorum*. A 100% rate of mycelial growth inhibition was gained by the application of 20 mg CNPs/mL. The antitumor activity of the green synthesized CNPs was examined using 6 different cell lines, the viability of the cells reduced when the concentration of green synthesized CNPs increased, the IC_50_ dose of the green synthesized CNPs on the examined cell lines HePG-2, MCF-7, HCT-116, PC-3, Hela and WI-38 was 36.25 ± 2.3, 31.21 ± 2.2, 67.45 ± 3.5, 56.30 ± 3.3, 44.62 ± 2.6 and 74.90 ± 3.8; respectively.

## Introduction

Chitosan is a white, rigid, and inelastic nitrogenous polysaccharide^1^. It is produced by deacetylating chitin, which is the primary component of crustacean exoskeletons. Chitosan is used in numerous applications; including food industry, biomedical industries, agriculture, paper manufacture, water treatment and environmental pollution control^2^. Chitosan also showed antifungal action against *Rhizopus stolonifer*, *Phomopsis asparagi*, *Rhizopus oryzae*, *Alternaria alternata*, and *Aspergillus niger* in its free polymer form^3^.

The chitosan and CNPs characteristics, including surface, small size, interface and quantum size effects, are combined in CNPs^4^. CNPs are natural polymers with exceptional physicochemical, antibacterial, and biological features, making them a superior ecological friendly material^5^. Chitosan, and chitosan nanoparticles, provide several advantages due to their improved stability, low toxicity, easy and mild synthesis method^6^.

Also, several researches have referred to the efficacy of chitosan nanoparticles as novel therapeutic strategies against viral infections^7^. CNPs are used for immunological prophylaxis, controlled-release drug delivery, and gene transfer in artificial organs^8^. Chitosan nanoparticles were utilized to produce a sustained release while applying sulphate of minoxidil to hair follicles without skin exposure^9^. Furthermore, chitosan nanoparticles were utilized to deliver herbicides for weed control^10^, in insecticide^11^, nanofertilizer for sustainable plant nutrition^12^ and fungicide treatment^13^. Although, CNPs show effective antimicrobial properties against pathogenic multidrug-resistant bacteria, *Acinetobacter baumannii*, *Staphylococcus aureus*, *Klebsiella pneumoniae*, *Pseudomonas aeruginosa*, and *Escherichia coli*^14,15^.

Nanoparticles could be synthesized by using wide variety of synthetic methods; physical, chemical, and biosynthetic routes which are considered particularly popular^16^. High pressure, energy, and temperature, as well as the presence of dangerous chemicals and huge particle sizes, all are limitations of both chemical and physical methods^17–19^. Nguyen et al.^20^ mentioned that the dimensions of chitosan nanoparticles produced via TPP ionic gelation and spray drying ranging between 166 and 1230 nm. Ionic gelation of tripolyphosphate and chitosan solution generated CNPs with dimensions ranging from 300 to 750 nm, according to Ha et al.^12^. Ghormade et al*.*^21^ established that the usual size of nanoparticles utilized in agricultural fields as nanofertilizers, nanoherbicides, and nanopesticides ranged from 100 to 500 nm. Nanoparticles should not exceed 100 nm in size, in order to reach tumor tissues, as; size, shape and surface characteristics have an important role in tumor targeted drug delivery, intermediate diameters (20–200 nm) have the strongest potential for *in-vivo* applications^22^. Therefore, it is crucial to create ecologically sustainable methods for the production of ultrafine nanoparticles.

The biosynthetic pathway is a green safe way of synthesizing nanoparticles from plants and microorganisms. It is biocompatible, and environmentally friendly for biomedical applications^23^. The green synthesis of nanoparticles was achieved using microorganisms, including algae. pigments^24,25^, algal derived soluble polysaccharides^26^, fungi^27^, and bacteria^28–31^. This synthesis can be performed by plants due to the presence of phytochemicals in the extract of certain plant parts (fruits, leaves, stem, seeds, and roots) which act as reducing and capping or stabilizing agents, to synthesize different nanoparticles^32^. The main objective of green synthesis is to decrease the use of toxic chemicals. Ali et al.^33^ reported that biological materials are suitable for use safely. CNPs appears as a promising candidate for a variety of applications, including the pharmaceutical industries, agriculture, medical applications, and biomedical engineering^14,34–37^.

Currently, there are around 20 species in the genus *Fusarium*^38^ Many species of *Fusarium* can cause *Fusarium* head blight (FHB) in wheat, one of the most significant fungal diseases globally^39^. A major factor in many regions throughout the world of *Fusarium* crown rot (FCR) and *Fusarium* root rot (FRR) of cereals is *Fusarium graminearum* along with *Fusarium pseudograminearum* and *Fusarium culmorum*^40^. *Fusarium* species can synthesize several mycotoxins throughout the infection process, particularly trichothecenes being the most important^41^. *Fusarium culmorum* is capable of producing mycotoxins of type B trichothecene deoxynivalenol (DON). DON is regarded as a factor of aggressiveness for wheat infection.

Lemongrass (*Cymbopogon citratus*) is a grass-family plant that originated in the warm climates of Asia's subtropics and tropics and cultivated in South and Central America, Africa, and other tropical regions^42^. The *Cymbopogon citratus* leaf provides an abundance of essential oil^43,44^. It includes citral, citronellol (cymbopogone and cymbopogonol), genariol, α-oxobisabolene and mycrene.

Considering the demand for environmentally friendly methods to produce chitosan nanoparticles, this study aims to synthesize CNPs using plant extract, to optimize the bio-synthesis process, to characterize the obtained CNPs, to evaluate their antifungal activity against phytopathogenic *Fusarium culmorum*, as well as evaluate their cytotoxic effects on various cell lines.

## Material and methods

### Preparation of the plant extract

*Cympopogon citratus* fresh leaves were gathered from Northern West Nile Delta, Wadi El-Natrun region, Egypt, approximately 100 kms from Cairo. The plant was graciously identified by Professor M. Azzazy of the University of Sadat City's Botany Department. The *Cympopogon citratus* leaves were collected, with permission, in line with legislation and institutional, national, and international standards. To eradicate contaminants, the leaves rinsed 3 times with distilled water. Then, 25 grammes of cleaned and refined chopped leaves were added to a flask containing 100 ml of distilled water, and then boiled for ten minutes^14^. After boiling, the solution was filtered using filter paper. The extract of *Cympopogon citratus* leaves was used for production of chitosan nanoparticles.

### Preparation of chitosan nanoparticles

Chitosan purchased from Bio Basic Inc. in Toronto, Canada, was mixed with acetic acid at a concentration of 1% (w/v), and then pH was adjusted to 5.0 with 1N NaOH after stirring for 24 h. Chitosan solution and the plant extract were mixed in equal quantities (10 mL for each) then shacked at 110 rpm and incubated at 50°C for thirty minutes. The obtained solution was centrifuged at 10,000 × g for ten minutes, washed, and then freeze-dried.

### Optimization of the green synthesized chitosan nanoparticles via Face  Centered  Central  Composite  Design (FCCCD).

Design Expert Software (version 12.0, https://www.statease.com/software/design-expert/) was used to create a four-variable FCCCD with 6 central runs for this study. FCCCD was used to determine each variable's optimal value for production of the highest yield of green synthesized chitosan nanoparticles using an aqueous extract of *Cympopogon citratus* plant leaves. The following were the four variables used: incubation time, temperature, initial pH and chitosan concentration. Each variable was evaluated using 3 coded levels (− 1, 0 and 1)^45^. The actual and coded levels, and the full matrix of the experimental design for variable levels. The response values (Y) for green synthesis of chitosan nanoparticles in each trial determined as the triplet's average. The following second-degree polynomial equation was used to determine the correlation between the selected independent variables and the response:

1 $$Y = \beta_{0} + \mathop \sum \limits_{i} \beta_{i} X_{i} + \mathop \sum \limits_{ii} \beta_{ii} X_{i}^{2} + \mathop \sum \limits_{ij} \beta_{ij} X_{i} X_{j}$$

In which ${X}_{i}$ correspond to the coded levels of the independent variables with Y being the predicted response, ${\beta }_{0}$ referred to a regression coefficient, ${\beta }_{i}$ represents the linear coefficient of determination ${\beta }_{ii}$ represents the quadratic coefficient, while ${\beta }_{ij}$ corresponds to the interaction coefficient.

### Statistical analysis

Design-Expert software (Version 12.0, Stat-Ease, Inc., Minneapolis, MN, USA) (https://www.statease.com/software/design-expert/), was used to generate the FCCCD design, to acheive an ANOVA (analysis of variance), the *P*-value, the *F*-value, and confidence levels, determination coefficient R^2^, and adjusted R^2^. STATISTICA (Version 8, StatSoft, Inc., Tulsa, USA) (https://www.statsoft.de/de/software/statistica) was used to create three-dimensional surface plots.

### Characterization of CNPs

#### UV–Visible spectra of the green synthesized CNPs

As a preliminary step in confirming the presence of nanoparticles, UV–visible spectra were recorded using an Optizen Pop-UV/Vis spectrophotometer. To verify the formation of nanoparticles, the solution was scanned between 200 and 400 nm.

#### Zeta potential

Malvern analytical Zeta sizer software version 7.13 was used to determine the value of Zeta potential of green synthesized CNPs, which was equipped with a laser doppler and identified with a phase analysis light scattering at 25°C and a Count Rate (kcps) of 172.8 with the samples in a liquid state.

#### Scanning electron microscope (SEM) investigation of CNPs

The shape and size of the green synthesized chitosan nanoparticles were studied using a 20 kV field emission scanning electron microscope (model JEOL-JSM-IT200) at Alexandria University, Faculty of Science, Alexandria, Egypt.

#### TEM (transmission electron microscope)

The TEM (JEM-2100 Plus, JEOL Ltd., Japan) at the City of Scientific Research and Technological Applications in Alexandria, Egypt, was used to assess the particle size and shape of the chitosan nanoparticles.

#### Energy dispersive X-ray (EDX) spectroscopy analysis

Energy dispersive X-ray Spectroscopy (TEM − EDX) confirmed the elemental composition of the synthesized nanoparticles, while FE-TEM EDX offered a quantitative explanation of the material's composition. Also, a mapping analysis was successfully carried out with TEM to determine the distribution and composition of synthesized CNPs.

#### XRD pattern

The X-ray diffraction patterns of the chitosan nanoparticles were obtained by a Bruker D2 Phaser 2nd Gen diffractometer. The source of the X-rays was Cu radiation with Kα1 ¼ 1.54060Ǻ (30 kV, 10 mA). Samples were scanned at a temperature of 25.7 °C with a diffraction angle of 2*θ* = 5–50 and a scanning rate of 2°/min^46^.

#### Differential scanning calorimetry (DSC) analysis

The thermal behavior of CNPs was studied using DSC analysis; sample (4.1 mg) was loaded on a standard aluminum pan, crimped, and heated from 25°C to 300°C at an average rate of 10°C per minute under continuous nitrogen purging (30 mL/min^-1^)^47^.

#### Thermo gravimetric analysis (TGA)

TGA was evaluated using temperature or time with a Model 50-H Thermo-Analyzer to measure the weight change of the green synthesized chitosan nanoparticles. 5.255 mg freeze-dried sample was loaded in the TGA furnace. The analysis was carried out in a nitrogen atmosphere, the heating rate of 40°C/min is maintained between 25 and 800°C.

#### Fourier transform infrared (FTIR)

FTIR spectroscopy with a Shimadzu FTIR-8400S was applied to validate the functional groups of the green synthesized chitosan nanoparticles. For analysis, potassium bromide (KBr) was mixed with dried CNPs. With a resolution of 1 cm^-1^, over a wave number range of 450 cm^-1^ to 500 cm^-1^, each KBr disc was scanned.

### Inhibition rate percentage of radial mycelial growth

The *Fusarium culmorum* strain used in this study was kindly provided and identified by Mona Youssry Diab Assistant lecture at faculty of agriculture, Department of Plant Pathology, Alexandria University, Egypt. The phytopathogenic fungus (*F. culmorum*) was grown on Potato Dextrose Agar (PDA) medium (200 g potatoes, 20 g glucose, and 15 g agar). From a 7-day-old culture of the test pathogens, a uniformly sized mycelial piece from the peripheral end (5 mm in diameter) was obtained and put down in the middle of the test Petri plates. All the Petri dishes were incubated at 28°C ± 1°C for 7 days, when control Petri dish cover full growth, the radial mycelial growth was recorded. The experiment was conducted twice, and each treatment included 3 replications. To determine the % inhibition rate of the pathogen's mycelia, the radial mycelial growth was compared to the control (without nanoparticles)^48^. % inhibition rate was calculated by the following equation:

2 $$\% {\text{Inhibition}}\;{\text{ rate}} = \left( {{\text{Mc}} - {\text{Mt}}} \right){\text{Mc}} \times 100$$

where Mt denotes the mycelial growth on the plates treated with different concentrations of CNPs solutions, and Mc indicates the mycelial growth on the control plate.

### In vitro antitumor activity test

Normal human lung fibroblast (WI38), human prostate cancer (PC3), colorectal cancer (HCT-116), hepatocellular carcinoma (HePG-2), epitheliod carcinoma (Hela), and mammary gland (MCF-7) cell lines were obtained from the Holding Company for Biological Products and Vaccines (VACSERA), Cairo, Egypt.

### MTT assay

Using the MTT assay, the inhibitory effects of the green synthesized chitosan nanoparticles were assessed on cell lines growth of WI38, Hela, HePG-2, MCF-7, HCT-116, and PC3. As a standard anticancer medication, doxorubicin was used^49,50^. The reagents RPMI-1640 medium, MTT and DMSO (sigma co., St. Louis, USA), Fetal Bovine serum (GIBCO, UK).

## Result and Discussion

This study focused on the green synthesis of CNPs using *Cympopogon citratus* leaves’ extract. Plant extracts play a crucial role in the green synthesis of chitosan nanoparticles. The plant extract is used as a linking material to bind with chitosan nanoparticles, resulting in the formation of the final product (CNPs)^35^. The biomolecules present in plant extracts, such as terpenoids, polyols, and polyphenols, act as reducing and capping agents for the nanoparticles^51^. The use of plant extracts in the synthesis of chitosan nanoparticles offers several advantages. Firstly, it is a renewable, non-toxic, biocompatible, and environmentally friendly method that does not harm the ecosystem^52^. Secondly, plant-based green synthesis is economically beneficial as it utilizes readily available and economically viable plant materials^53^ Additionally, different parts of plants, including roots, stems, leaves, and fruits, can be used for the synthesis process^54^. The green synthesis of chitosan nanoparticles using plant extracts has been shown to have effective antibacterial activity against various pathogens^14^.

**Figure 1:**
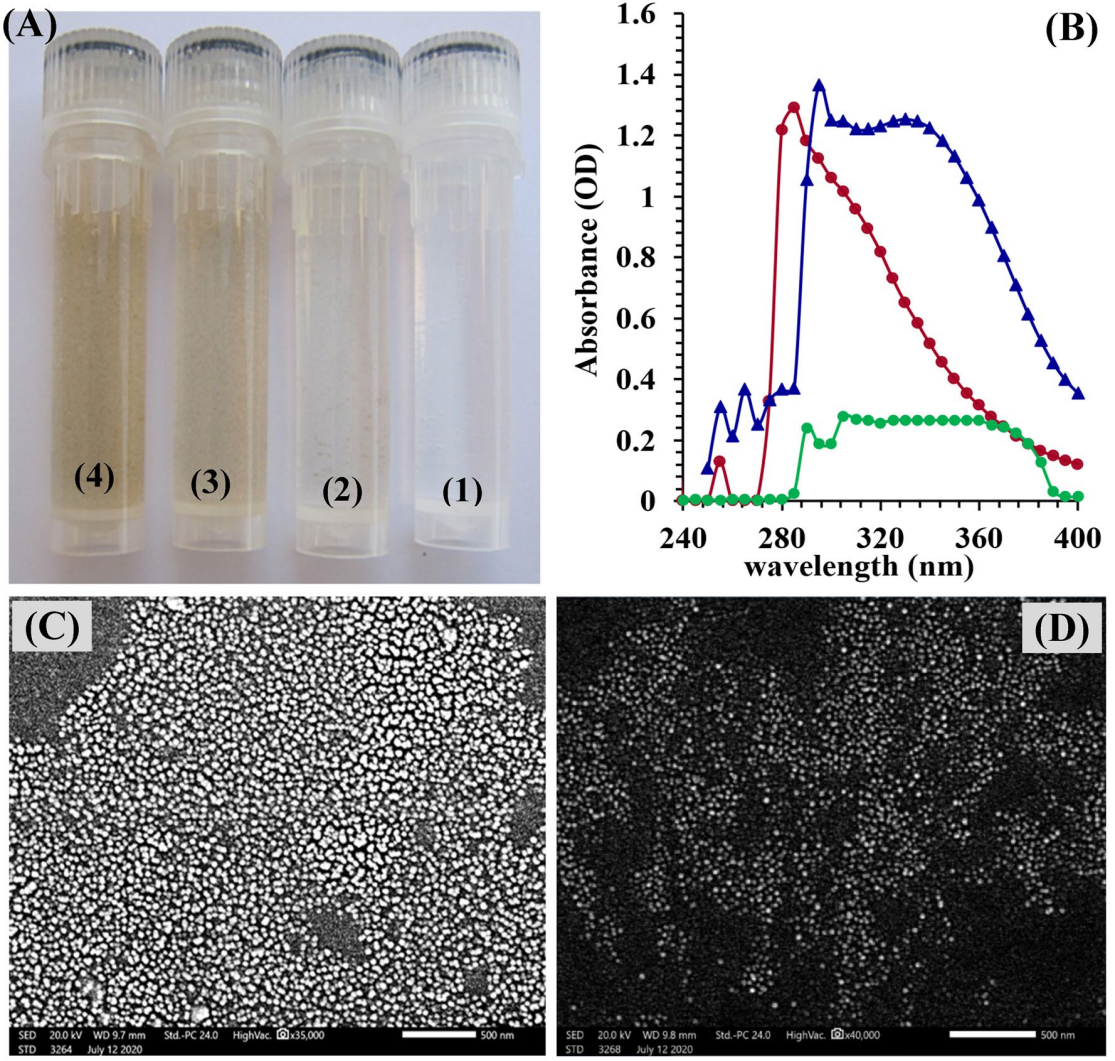
(**A**) vials contain (1, Chitosan solution; 2,3,4 biosynthesized chitosan nanoparticles with different incubation time; 10, 20 and 30 min.). (**B**) UV/visible spectra of chitosan (red line) and chitosan nanoparticles (blue line) and *Cympopogon citratus* extract (green line); (the maximum absorbance wavelength of CNPs at 295 nm). (**C**,**D**) Scanning electron microscope (SEM) micrographs.

Figure 1A vial number (1) show chitosan solution, while the green synthesized chitosan nanoparticles with different incubation times were shown as the following; vial number (2) shows green synthesized CNPs after 10 min of incubation, vial number (3) shows green synthesized CNPs after 20 min of incubation and vial number (4) shows green synthesized CNPs after 30 min of incubation. The UV–visible spectrum range of 200–400 nm was used to characterize the green synthesized CNPs, chitosan nanoparticles and *Cympopogon citratus* extract were shown in Fig. 1B. The maximum absorbance was observed at 295 nm, revealing peaks of CNPs.

### Characterization of the green synthesized chitosan nanoparticles

#### UV–visible spectrophotometric analysis

In order to find the absorbance peak, a UV/Vis spectrophotometer was utilized to conduct a wavelength range scan 200 to 400 nm of the green synthesized CNPs. The scan spectrum of chitosan (red line) and chitosan nanoparticles (blue line) and *Cympopogon citratus* extract (green line), shown in Fig. 1B, the maximum absorbance wavelength of CNPs was observed at 295 nm and the maximum absorbance wavelength of chitosan standard was observed at 285 nm. the UV/visible spectrum of chitosan nanoparticles is attributed to the substantial number of the biosynthesized nanoparticles. These results are similar with El-Naggar et al*.*^35^ study, which showed an absorption peak wavelength of 295 nm. The narrow peak at about 290 nm obtained from the *Cympopogon citratus* extract is mainly attributed to the UV absorption of polyphenols^55^. The *Cympopogon citratus* extract contained polyphenolic compounds that may be responsible for the synthesis of chitosan nanoparticles as reducing and stabilizing agent^56^. Chitosan after the addition of *Cympopogon citratus* extract, has shown a distinct shift of about 10 nm. this could be attributed to the formation of the CNPs. Chitosan's UV/Visible spectrum exhibited a broad absorption range; thus, the high strength level of CNPs biopolymer demonstrates that Phyto-fabrication is an efficient method for producing CNPs^57,58^.

#### Size and morphology of CNPs

Both scanning electron microscopy (SEM) and transmission electron microscopy (TEM) are useful tools for examining size and shape of the nanoparticles. SEM micrographs of the green synthesized CNPs are shown in Fig. 1C, D. Chitosan nanoparticles in the SEM image showed that the surface of the nanoparticles was smooth. The homogeneity of nanoparticles shape was observed across all samples. Khan mohammadi et al.^59^ mentioned that the chitosan nanoparticles produced exhibited an average particle size ranging from 33.64 to 74.87 nm, as examined using FE-SEM. whereas Asgari-Targhi et al*.*^60^ calculated the average nano-chitosan size to be 43.32 nm using FE-SEM.

**Figure 2:**
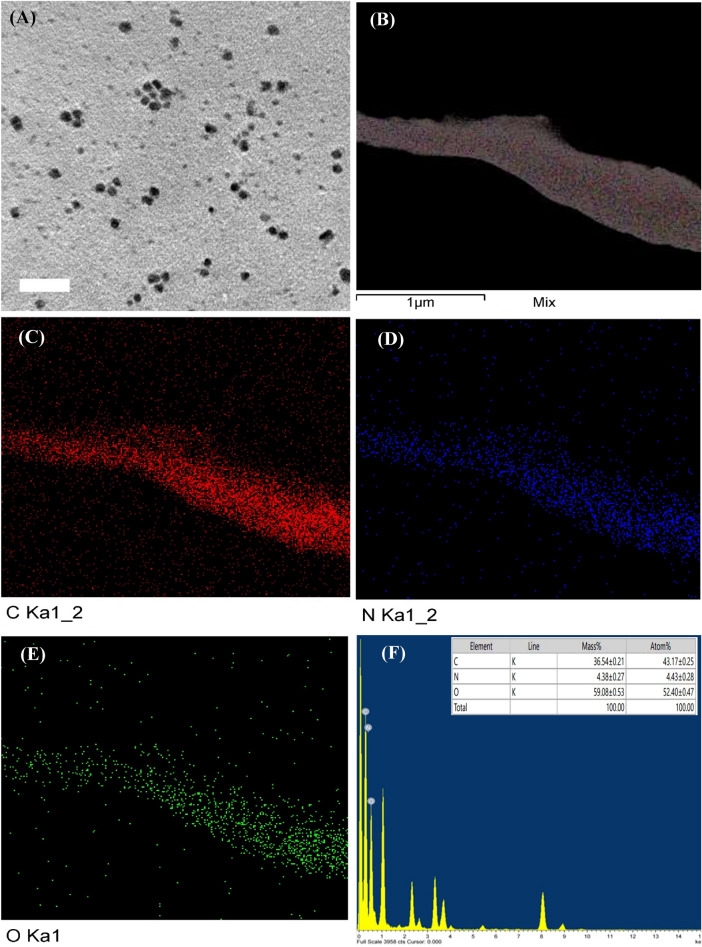
(**A**) Transmission electron microscope (TEM) micrograph, (**B**-**E**) Mapping analysis and (**F**) EDX analysis of green synthesized chitosan nanoparticles using *Cympopogon citratus* extract.

TEM micrograph of the green synthesized CNPs is shown in Fig. 2A. TEM image displays spherical nanoparticles with size range from 8.08 to 12.01 nm. Gadkari et al*.*^61^ mentioned that several spherical chitosan-cinnamaldehyde nanoparticles were detected with size range from 80 to 150 nm in TEM images. While in Mubarak-Ali et al*.*^62^ study, TEM images showed spherical CNPs with 200 nm diameter. Also, Alqahtani et al*.*^63^ revealed that TEM images of CNPs display a spherical shape, a smooth surface, and size range from 150 to 336 nm. Green synthesized CNPs have the smallest particle size compared to other studies mentioned previously.

Micrographs of the dried green synthesized CNPs by TEM mapping are shown in Fig. 2B-E. It was observed that CNPs tended to aggregate and had relatively uniform sizes. Moreover, the mappings of C, N, and O elements exhibited even distribution, which demonstrated that the CNPs were formed by using *Cympopogon citratus* leaves extract in an equal manner.

#### EDX analysis

Energy-dispersive X-ray spectroscopy (EDX) analysis was used to investigate the chemical composition and principal constituents of green synthesized CNPs by using *Cympopogon citratus* leaves extract (Fig. 2F). In addition, the elemental mapping of EDX patterns revealed the presence of the carbon (C), nitrogen (N), and oxygen (O) with atom percentage of 43.17 ± 0.25%, 4.43 ± 0.28% and 52.40 ± 0.47%; respectively. EDX analysis of CNPs synthesized by *Lavendula angustifolia* leaves extract revealed that the obtained CNPs contain nitrogen, oxygen and carbon^64^.

#### Fourier transforms infrared (FTIR) measurements

**Figure 3:**
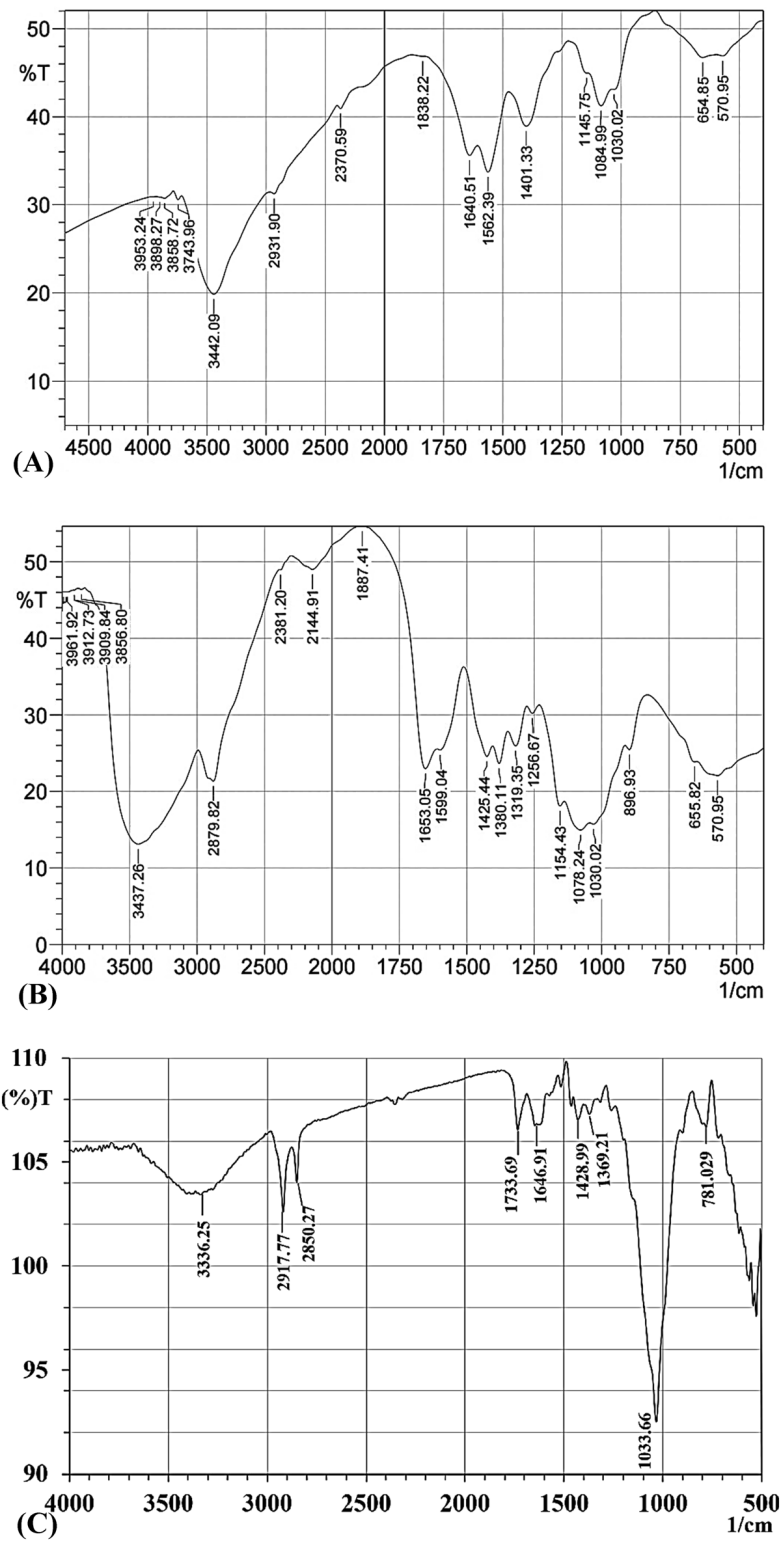
(**A**) FTIR analyses for green synthesized chitosan nanoparticles using *Cympopogon citratus* extract, (**B**) chitosan standard solution and **C**) *Cympopogon citratus*.

In terms of chemical structure, FT-IR spectroscopy was used to characterize the functional groups. The FTIR spectra of chitosan, chitosan nanoparticles and *Cympopogon citratus* aqueous extract were investigated. Significant shifts in the peaks of the FTIR spectrum of CNPs when compared to the peaks of the standard chitosan and *Cympopogon citratus* extract. The changes of the IR spectra of chitosan plant extract membranes due to the synergistic effect of phenolic compounds in the extracts and the interactions between them and the chitosan chain structure^65^. Table 1 and Fig. 3 illustrate the Fourier transforms infrared spectrum for A) green synthesized chitosan nanoparticles, B) chitosan standard solution and D) *Cympopogon citratus* extract in the range of 4000–500 cm^-1^. The first group of bands appeared in the spectra between 4057 and 3750 cm^-1^, indicating the combination of functional groups of –NH2, –CH, C–C, and –OH^36^. The peak at 3743  cm^-1^ in CNPs spectrum is attributed to N–H stretching vibrations^66^. The presence of bands at 3437 cm^−1^ in chitosan standard sample indicate strong dimeric O–H stretching^67^, this peak was found in the spectrum of chitosan nanoparticles shifted to 3442.09 cm^−1^ which indicates the stretching vibrations of OH groups^68^. In the FTIR spectrum of *Cympopogon citratus* extract a band at 3336.25 cm^−1^ was noticed, which shows the presence of strong phenolic compound^69^. An absorption band at 2917.77 cm^−1^ were found in *Cympopogon citratus* extract spectrum can be attributed to C-H symmetric stretching, this band has polysaccharide properties^70^. The same band appeared in CNPs spectrum the peak was shifted to 2931 cm^-1^ which corresponds to C-H group stretching^71^.

**Table 1:** FTIR peaks of  the green synthesized chitosan nanoparticles with the annotations and reference of each peak.

No	Wave no. (cm^−1^)	Annotations	References
1	570	C=C of benzene ring	Hirpara et al.^85^
2	∼655 (654.85)	Corresponds to N–C = O	Abdelghany et al.^84^
3	1030	C-O stretch	Azhary et al.^82^
4	1084	C-O stretching vibration	Cahyana et al.^79^
5	1145	C-S stretching vibrations of sulfides and disulfides	Farhadian et al.^78^
6	1401	N–H stretching of amide and ether bonds	Das et al.^77^
7	1562	N–H bending vibration	Li et al.^76^
8	1640	N–H deformation	Safari et al.^74^
9	2370	CN group	Byeon et al.^72^
10	2931	C-H stretching	Swarnalatha et al.^71^
11	3442	OH group stretching vibration	de Pinho Neves et al.^68^
12	3743	N–H bending vibrations of primary amine	Prayag et al.^66^

In addition, the presence of a peak at 2370 cm^-1^ corresponding to the -CN group confirmed the incorporation of succinyl groups on the chitosan backbone^72^. Bands at 2144.91, 1380.11 and 1319.35 cm^−1^ disappeared in the FTIR spectrum after CNPs green synthesis, reveals that these groups are involved in the CNPs green synthesis by *Cympopogon citratus* aqueous extract. Krishnaveni and Ragunathan^67,73^ reported that band at 1653.05 cm^−1^ (around 1655 cm^−1^) indicate the stretching vibration of type I amide. The intense peak of amide I region shifted from FTIR spectrum of chitosan standard to 1640 cm^−1^ in FTIR spectrum of CNPs which represents N–H deformation^74^, while the band at 1646.91 cm^−1^ in *Cympopogon citratus* extract FTIR spectrum, indicating interactions between protonated amine groups of the chitosan standard with the components of the plant extract of *Cympopogon citratus*. In chitosan nanoparticles, this peak is sharper and shift toward 1640 cm^−1^ indicating increased interactions or bonding^36^. So, the shifting of vibrations from higher to lower wave number reveals the formation of CNPs^75^.

The peak at 1562 cm^-1^ is associated with N–H bending vibration^76^. N–H stretching of amide and ether bonds induces a peak close to 1401 cm^−1^ ^77^. In FTIR spectrum of chitosan standard, amide III region presence was indicated by band at 1380 cm^−1^ ^75^. Bands at 1256.67 and 896 cm^−1^ appeared in standard chitosan spectrum and bands at 1733.69 and 1369.21 cm^−1^ appeared in *Cympopogon citratus* extract spectrum, all of these peaks were disappeared in the FTIR spectrum after CNPs green synthesis, reveals that these groups are involved in the CNPs green synthesis by the *Cympopogon citratus* aqueous extract. FTIR spectrum of CNPs showed both 1145 cm^−1^ and 600 cm^−1^ peaks, which were related to the C-S stretching vibrations of sulphides and disulfides^78^. Moreover, the peak seen at 1084 cm^−1^ is typical of the C-O stretching vibration in CNPs^79^. *Cympopogon citratus* extract FTIR spectrum reveled a peak at 1033.66 cm^-1^ which corresponds to the symmetric and asymmetric stretching vibration of C-O-C^ 80,81^. In the FTIR spectrum of CNPs, the peak at 1030.02 cm^−1^ is attributed to the C-O stretching vibration^81,82^.

The small peaks found at the end of the FTIR spectra correlate to the looping of the saccharide structure of chitosan^35,83^. In CNPs FTIR spectrum, the peak observed at a wavenumber of ∼655 cm^−1^ (at 654.85 cm^−1^) corresponds to N–C = O^84,35^. The peak at 570.95 cm^-1^ attributed to the absorption band of the benzene ring's out-of-plane C = C bonding this peak in CNPs^85^.

FTIR analysis declares the occurrence of capping groups to the surface of CNPs, which stabilize the CNPs, as well as prevent their coagulation and/or aggregation in the colloidal phase^36^.

#### Zeta potential

**Figure 4:**
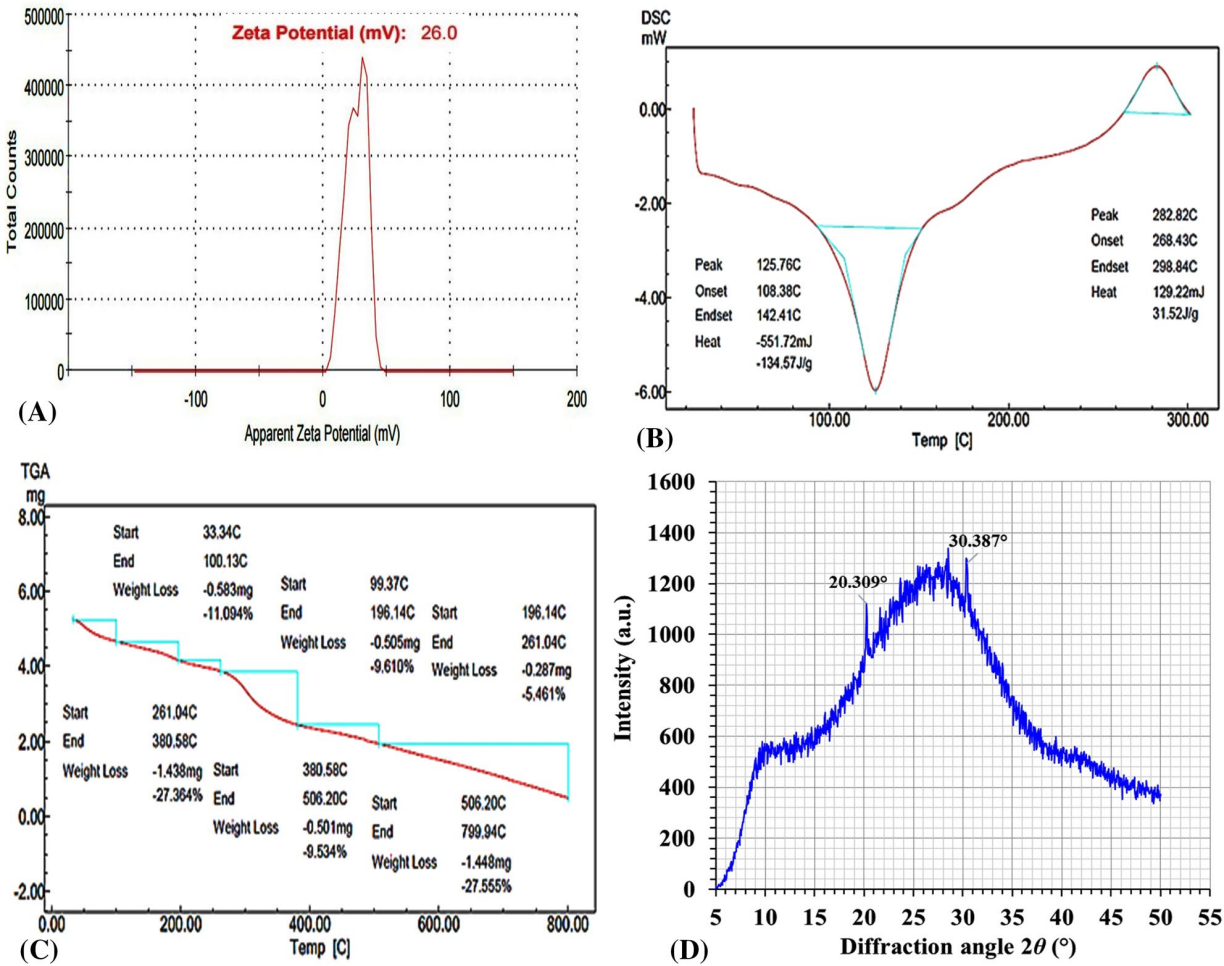
(**A**) Zeta potential, (**B**) DSC, (**C**) TGA, and (**D**) XRD analyses of green synthesized chitosan nanoparticles using *Cympopogon citratus* extract.

The positive charge as indicated by the Zeta potential value is essential for maintaining the stability of the colloidal solution^86^. The Zeta potential of the green synthesized CNPs is + 26 mV (Fig. 4A) and has a narrow range, which means an increase in surface area, an increase in dispersion capacity, and an improvement in catalytic activity^87^.

The amino group of chitosan is attributed to its positive Zeta potential, strong positive charge is required to prevent accumulation^88^. Zhang et al.^89^ mentioned that the samples of CNPs had a Zeta-potential that varied from -0.54 to + 35.97. Due to electric repulsion, nanoparticle aggregation decreased and Zeta potential increased, showing strong physical stability of the synthesized nanoparticles. In Khan et al.^90^ study the Zeta potential was + 31 ± 3.14 mV, while Asal et al.^91^ discovered that the Zeta potential of chitosan nanoparticles synthesized was + 31 ± 2.2 mV. As reported by Raza and Anwar^92^, it was determined that the Zeta potential on the surface of CNPs is + 31.3 mV. El-Naggar et al*.*^35^ reported that the Zeta potential was about + 32.6 ± 5.26 mV which indicates relative stability at 25°C for positively charged chitosan nanoparticles, moreover, the single peak in the Zeta potential distribution shows that CNPs are distributed uniformly. In contrast, when the Zeta potential is low, force of attraction may increase, causing flocculation attributable to the dispersion's disintegration. Thus, colloids exhibiting a significant positive or negative Zeta potential possess a greater charge. stable than those with a low Zeta potential and tend to agglomerate^93^. It was shown in Yien et al.^3^ study that the inhibitory impact of chitosan nanoparticles depended on both particle size and Zeta potential, which was observed from + 22 to + 55 mV.

#### Differential scanning calorimetry (DSC)

Figure 4B shows the use of differential scanning calorimetry (DSC) for studying the thermal behavior of green synthesized CNPs. The first endothermic peak ranged from 108.38°C to 142.41°C. While the second exothermic peak ranged from 268.43°C to 298.84°C. CNPs vary in their water-holding capacity under varied pH condition, which correlates to bound water evaporation from samples^89^. The loss of water from hydrophilic groups is related to an endothermic peak at 100°C, while the degradation due to dehydration and depolymerization was determined with an exothermic peak at 306.0°C^88^.

#### Thermogravimetric analysis (TGA)

The TGA of the green synthesized CNPs exhibits weight loss in six phases, as shown in Fig. 4C, the first stage involved weight loss of 11.094% between 33.34°C and 100.13°C. This may be related to the reduction of moisture content and a greater quantity of water loss due to various configurations of molecular chains in amorphous domains. The second phase starts at 99.37°C and goes up to 196.14°C. There is 9.610% weight loss. While increasing the temperature over 300°C, as in the fourth stage (27.364% weight loss), CNPs were damaged, resulting in a considerable weight loss^76^. The fifth stage from 380°C to 506°C results in weight loss of 9.534%. However, the sixth stage from 506.20°C to 799.94°C caused a weight loss of 27.555%. TGA peaks reflected mass loss at corresponding temperatures of 100.29°C (11.78%), peak 245.43°C (19.21%), 280.27°C (37.59%), 525.60°C (51.86%), and 579.68°C (75.40%), respectively, as reported by Sethi et al*.*^94^. While Mahmoud et al*.*^95^ discovered that the TGA of chitosan nanoparticles exhibits degradation phases at 38.88–180.48, 180.48–267.02 and 267.02–599.30 °C, with percentage loss values of 13.97, 21.62 and 14.92%, respectively. The partial heat decomposition of chitosan nanoparticles is mostly responsible for these three stages of degradation.

#### X-ray diffraction (XRD) analysis

The crystalline structure of green synthesized chitosan nanoparticles was analyzed using XRD. Amorphous structure is demonstrated by the XRD analysis of CNPs^96^. Chitosan nanoparticles displayed peaks in the 2θ range of 10–40 at 23.7°C, 10 mA, and 30 kV. As shown in Fig. 4D, the CNPs sample exhibited 2 distinct 2θ peaks on its XRD pattern at 20.309 and 30.387°. Representing a deviation from the typical chitosan peaks which reveal broad peaks at 2θ = 10° and 2θ = 20°^97^. XRD patterns with a prominent peak at an angle of 20.309° revealed that chitosan nanoparticles have a crystalline structure.

In accordance with our findings, the pattern of CNPs sample in El-Naggar et al.^14^ study revealed three unique 2θ peaks, at 10.86, 20.74, and 30.78°, deviating from standard chitosan peaks. The crystalline structure of the nanoparticles was identified by XRD data, which displayed a conspicuous peak at an angle of 20.74°. While Budi et *al.*^98^ reported that peaks at 2θ of 10.18° and 20.26° were seen in XRD diffraction patterns of chitosan and chitosan nanoparticles. These diffraction peaks reveal the (020) hydrated crystalline form and the (110) anhydrous crystalline structure of chitin. It also indicates that chitosan nanoparticles have a crystalline phase. Yen et al*.*^99^ likewise discovered a chitosan peak at 2θ = 20.7°. Chitosan's highly crystalline structure is suggested by the presence of both weak and strong diffraction peaks at diffraction angle 2θ = 11.9° and 2θ = 20°, respectively^100^.

### Face-centered central composite design (FCCCD) optimization

The optimum levels and interactions of the following variables were determined using (FCCCD): incubation time (min), temperature (°C), starting pH level, and chitosan concentration (%). This research had 30 trials with various combinations of chitosan concentration (A), initial pH level (B), temperature (C), and incubation period (D) were conducted, and the impacts of four independent factors on the green synthesis of chitosan nanoparticles, together with the predicted response and residuals, are shown in Table 2. The outcomes of eco-friendly chitosan nanoparticle synthesis exhibited a wide range of variance. The maximum production of green synthesized CNPs was obtained in run number 8 (13.99 mg CNPs/mL) with the following conditions: chitosan concentration: 1.5%, pH: 4.5, temperature: 40 °C, and incubation period: 30 min. The lowest production of green synthesized CNPs (5.57 mg CNPs/mL) appeared in run number 5 under the conditions of chitosan concentration: 2%, pH: 5, temperature: 40 °C, and incubation period: 10 min.

**Table 2:** FCCCD matrix presenting green synthesis of chitosan nanoparticles by *Cymbopogon citrates* extract affected by  chitosan concentration (A), initial pH level (B),  temperature (C), and  incubation period (D).

Std	Run	Type	A	B	C	D	Chitosan nanoparticles (mg/mL)		
							Actual	Predicted	Residuals
7	1	Fact	− 1	1	1	− 1	9.33	9.38	− 0.05
9	2	Fact	− 1	− 1	− 1	1	8.9	8.88	0.02
29	3	Center	0	0	0	0	13.63	12.87	0.75
23	4	Axial	0	0	0	− 1	12.69	12.38	0.31
4	5	Fact	1	1	− 1	− 1	5.57	5.62	− 0.05
8	6	Fact	1	1	1	− 1	7.74	7.73	0.01
16	7	Fact	1	1	1	1	10.13	9.96	0.17
21	8	Axial	0	0	− 1	0	13.99	13.61	0.38
15	9	Fact	− 1	1	1	1	9.68	9.7	− 0.02
13	10	Fact	− 1	− 1	1	1	6.62	6.69	− 0.07
12	11	Fact	1	1	− 1	1	9.68	9.7	− 0.02
14	12	Fact	1	− 1	1	1	11.99	12	− 0.01
11	13	Fact	− 1	1	− 1	1	9.88	9.94	− 0.06
28	14	Center	0	0	0	0	12.14	12.87	− 0.73
2	15	Fact	1	− 1	− 1	− 1	12.36	12.31	0.05
19	16	Axial	0	− 1	0	0	12.12	11.73	0.39
1	17	Fact	− 1	− 1	− 1	− 1	9.11	9.4	− 0.29
18	18	Axial	1	0	0	0	11.83	11.85	− 0.02
20	19	Axial	0	1	0	0	9.86	9.89	− 0.03
27	20	Center	0	0	0	0	13.47	12.87	0.60
10	21	Fact	1	− 1	− 1	1	13.63	13.7	− 0.07
17	22	Axial	− 1	0	0	0	10.66	10.27	0.39
3	23	Fact	− 1	1	− 1	− 1	7.8	7.76	0.04
6	24	Fact	1	− 1	1	− 1	12.42	12.48	− 0.06
24	25	Axial	0	0	0	1	13.29	13.23	0.06
26	26	Center	0	0	0	0	12.24	12.87	− 0.64
22	27	Axial	0	0	1	0	13.56	13.57	− 0.01
30	28	Center	0	0	0	0	12.09	12.87	− 0.78
25	29	Center	0	0	0	0	12.58	12.87	− 0.29
5	30	Fact	− 1	− 1	1	− 1	9.11	9.07	0.04

Variable	Code	− 1	0	1					
Chitosan conc. (%)	A	1	1.5	2					
Initial pH level	B	4	4.5	5					
Temperature (°C)	C	40	50	60					
Incubation period (min)	D	10	30	50					

### Analysis of multiple regression and ANOVA

For the model's reliability, the calculations in Table 3, such as the estimated coefficient values, the determination coefficient (R^2^) value, Adj. R^2^ value, predicted R^2^ value, *F*-value (Fisher value), *P*-value (probability value), and lack of fit, have been evaluated. Additionally, the linear, interaction, and quadratic effects of the four selected process factors was determined^101^. The present model has an R^2^ value of 0.9779. A high level of correlation was considered to be achieved when the model's (R^2^) value is more than 0.9^25^. When the R^2^ value is relatively close to 1, the model is more accurate in predicting the response^102^. Our analysis suggests that the model used for CNPs green synthesis has an R^2^ value of 0.9779, reflecting that 97.79% of the variance in the green synthesized CNPs was assigned to independent factors. However, the model can not only explain 2.21% of the overall variance. The adjusted determination coefficient for the regression model of the green synthesized CNPs is shown in Table 3 (Adj R^2^ = 0.9573); a higher value suggests greater significance^103^. The predicted R^2^ value of 0.9564 and the Adj. R^2^ value showed that the observed and predicted values of the response were very similar^104^. Table 3 displays the mean, standard deviation, and adequate precision of the model as 10.94, 0.47, and 24.53, respectively. Appropriate precision indicates noise level; the level > 4 (24.53) is superior and reveals high accuracy, indicating an appropriate design space for optimizing green synthesis of CNPs at different ranges of studied parameters^64^. The statistical analysis of the green synthesized CNPs data reveals a coefficient of variation (C.V.) of 4.26%, which is quite low and indicates the high accuracy, reliability, and precision of experimental trials^64^.

**Table 3:** Analysis of variance for green synthesis of chitosan nanoparticles using *Cymbopogon citrates* extract influenced by chitosan concentration (%), initial pH level, temperature (°C) and incubation period (min).

Source of variance		Coefficient estimate	Sum of squares	Degrees of freedom	Mean Square	*F*-value	*P*-value
Model	Intercept	12.87	144.24	14	10.30	47.49	< 0.0001*
Linear effect	A	0.79	11.29	1	11.29	52.05	< 0.0001*
	B	− 0.92	15.30	1	15.30	70.51	< 0.0001*
	C	− 0.02	0.01	1	0.01	0.03	0.8642
	D	0.43	3.27	1	3.27	15.05	0.0015*
Interaction effect	AB	− 1.26	25.57	1	25.57	117.87	< 0.0001*
	AC	0.12	0.25	1	0.25	1.15	0.3015
	AD	0.48	3.62	1	3.62	16.69	0.001*
	BC	0.49	3.81	1	3.81	17.58	0.0008*
	BD	0.67	7.28	1	7.28	33.56	< 0.0001*
	CD	− 0.46	3.45	1	3.45	15.89	0.0012*
Quadratic effect	A^2^	− 1.81	8.51	1	8.51	39.23	< 0.0001*
	B^2^	− 2.07	11.08	1	11.08	51.05	< 0.0001*
	C^2^	0.72	1.34	1	1.34	6.16	0.0254*
	D^2^	− 0.07	0.01	1	0.01	0.05	0.8187
Error effect	Lack of Fit		0.89	10	0.09	0.19	0.9877
	Pure Error		2.36	5	0.47		
R^2^	0.9779	Std. Dev	0.47				
Adj R^2^	0.9573	Mean	10.94				
Pred R^2^	0.9564	C.V. %	4.26				
Adeq precision	24.53						

*Significant values.

In addition, the estimated coefficient revealed positive or negative effects on the green synthesis of CNPs. Whether it is positive or negative, a large estimated effect shows that the independent factors significantly affect the response. Production increases with high concentrations of any tested variable whose predicted effect has a positive sign. In contrast, a negative sign denotes increased production when the variable is at low concentrations^30^.

Two variables can interact in a synergistic (positive coefficient) or antagonistic (negative coefficient) manner. The two variables, A and D showed positive coefficients that these variables increased the green synthesis of CNPs in a linear manner. Additionally, negative coefficients for B, and C showed a linear relationship between these factors and cause reduction in the green synthesis of CNPs production. The interaction effect showed that AC, AD, BC, and BD increase the green synthesis of CNPs as they have a positive coefficient, while negative coefficients for AB, and CD. The quadratic effect reveals that A^2^, B^2^, and D^2^ have negative coefficient and only C^2^ that have a positive coefficient that cause increase in the green synthesis of CNPs production.

The significance of each coefficient was examined using probability values (*P*-values) and *F*-values (Table 3), which were required in order to evaluate the significance of the variables to understand their interactions. The coefficient's significance increased as the *P*-values decreased^37^. Additionally, process variables were considered to have a significant impact on the response when their *P*-values were less than or equal to 0.05^105^. The model had a high level of significance, as indicated by its *F*-value of 47.49 and *P*-value of < 0.0001. According to the *P*-values of the coefficient the linear effects of chitosan concentration (A), initial pH level (B), and incubation period (D) besides the interaction effect of chitosan concentration with initial pH level (AB), chitosan concentration with incubation period (AD), initial pH level with temperature (BC), initial pH level with incubation period (BD), and temperature with incubation period (CD) , as well as the quadratic effect of chitosan concentration (A^2^), initial pH level (B^2^), and temperature (C^2^) are significant for the environmentally friendly synthesis of CNPs using *Cympopogon citratus* leaves extract. Because of this, they act as limiting variables, and even slight changes in their concentrations will have an impact on the production of green produced CNPs.

The fit of summary results for choosing the polynomial model with the most appropriate model terms and an inadequate lack of fit is shown in Table 4. Additionally, the fit of summary data indicates which model has the lowest standard deviation, the highest adj. and pred. R^2^ values. The quadratic model has insignificant lack of Fit Test for green synthesized CNPs (*P*-value = 0.9877, *F*-value = 0.19). The quadratic model of green synthesized CNPs had also the lowest standard deviation, at 0.47, and the highest pred. and adj. R^2^ values, at 0.9564 and, 0.9573, respectively (Table 4). The following second-order polynomial equations describe the relationship between the chosen independent variables and CNPs:

**Table 4:** Fit summary for Face-centered central composite design results for green synthesis of chitosan nanoparticles using *Cymbopogon citrates* leaves extract.

Source	Sum of squares	*Df*	Mean square	*F-*value	*P-*value *P*rob > *F*
Lack of fit tests					
Linear	115.27	20	5.76	12.20	0.0057*
2FI	71.28	14	5.09	10.78	0.008*
Quadratic	0.89	10	0.09	0.19	0.9877
Sequential model sum of squares					
Linear vs Mean	29.86	4	7.47	1.59	0.2088
2FI vs Linear	43.99	6	7.33	1.89	0.1348
Quadratic vs 2FI	70.39	4	17.60	81.12	< 0.0001*
Source	Standard deviation	R-squared	Adjusted R-squared	Predicted R-squared	PRESS
Model summary statistics					
Linear	2.17	0.2025	0.0749	-0.2336	181.95
2FI	1.97	0.5007	0.2379	-0.7358	256.02
Quadratic	0.47	0.9779	0.9573	0.9564	6.43

*Significant values, *df*: degree of freedom, PRESS: sum of squares of prediction error, two factors interaction: 2FI.

The predicted value of green synthesized CNPs mg/mL = 12.87 + 0.79A – 0.98B – 0.02C + 0.43D – 1.26AB + 0.12 AC + 0.48AD + 0.49 BC + 0.67BD – 0.46CD – 1.81 A^2^ – 2.07 B^2^ + 0.72 C^2^ – 0.07 D^2^. Equation (3)

Where A‒D are the coded values of chitosan concentration, initial pH level, temperature, and incubation period; respectively.

There is an antagonistic relation between the production % and the variable(s) which is determined by the negative coefficient values, while the positive coefficient value indicates a synergistic relationship. Therefore, the negative values of quadratic, mutual, and linear effects of the specified process variables reveal that they negatively impacted the production of green synthesized CNPs percentage; green synthesized CNPs are produced at a higher rate when the coefficient values are positive within the evaluated range of the selected four parameters.

### Three-dimensional (3-D) surface plot of CNPs

**Figure 5:**
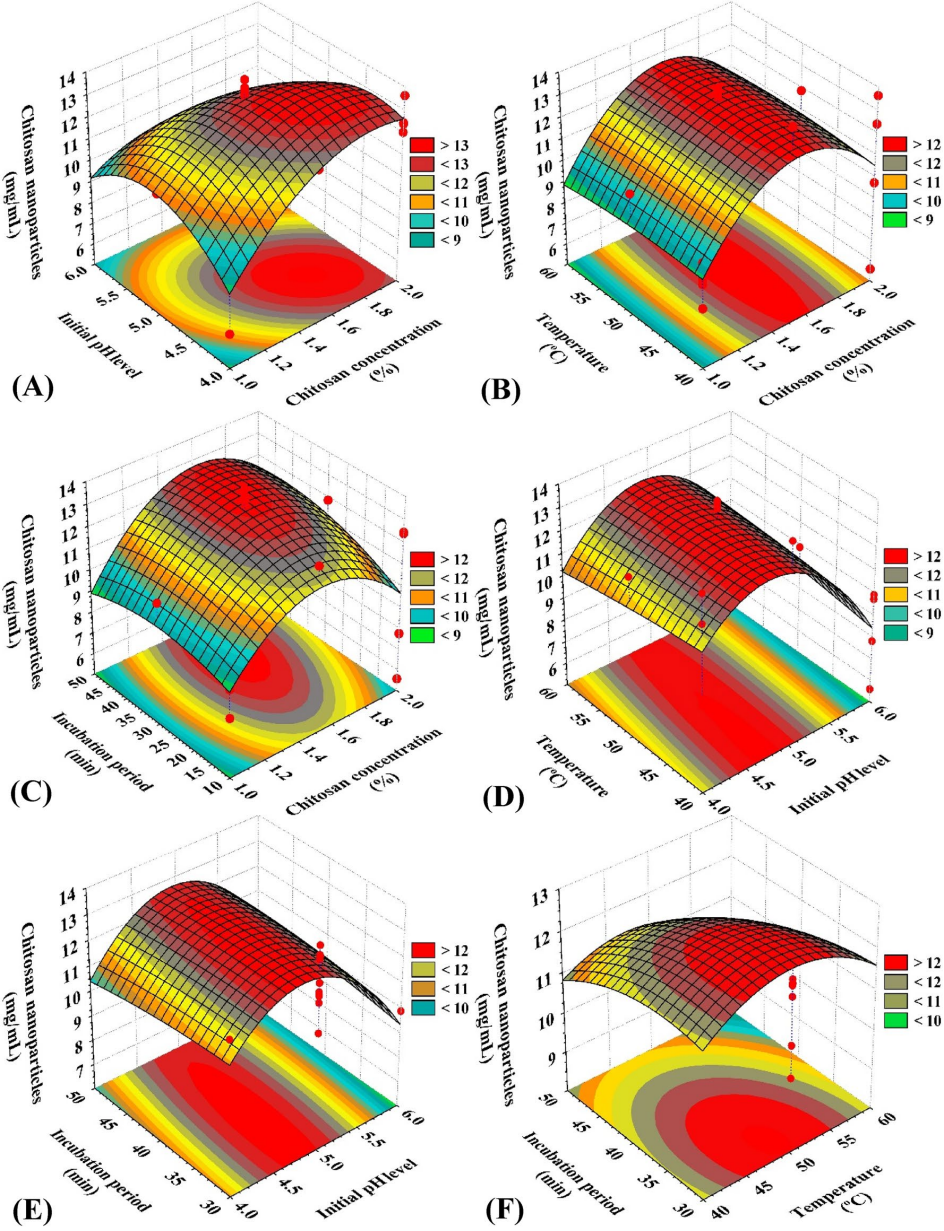
3-dimensional (3D) surface plot for the green synthesis of chitosan nanoparticles using *Cymbopogon citrates* extract.

The optimum conditions for the green synthesis of chitosan nanoparticles were determined with the use of 3D surface plots (Fig. 5), which were designed to display the correlations between the interactions of the designated process parameters and the responses. Four variables were combined in pairs (temperature, initial pH level, chitosan concentration, and incubation time) in order to generate three-dimensional graphs. two process parameters were plotted against the green chitosan nanoparticles production on the Z-axis, when other two process parameters held constant at their midpoints.

### Role of initial pH on the green synthesis of CNPs

The Fig. 5 A, D, and E are 3D surface graphs that show how the initial pH level affects green synthesis of CNPs when it interacts with the other 3 variables: chitosan concentration, temperature, and incubation time, respectively. The graphs show that when starting pH increased, the green synthesis of CNPs increased. In the middle of the initial pH range, the maximum CNPs green synthesis was observed (around 5.0). Green synthesis of CNPs decreased as a result of additional increase or decrease. As determined by Sathiyabama and Parthasarathy^57^, the best initial pH for maximum CNPs production was 4.8.

The best initial pH for maximum CNPs production, as determined by Sathiyabama and Parthasarathy^57^, was 4.8. Tang et al.^106^ discovered that the mean diameter of chitosan nanoparticles reduced when pH increased, suggesting that pH had an effect on particle size. This may be a result of the varying molecular conformation of chitosan that occurs prior to nanoparticles production as the pH changes. Due to the high repulsion between positively charged amino groups at low pH (3.0), the bulk of the amino groups in chitosan were protonated in acidic solution, forming an extended molecular chain (isoelectric point = 6.8)^42^. In the study by Ali et al*.* (2011)^107^, the average particle size does not fluctuate considerably over the pH range of 3.0 to 4.5. El-Naggar et al.^37^ mentioned that as initial pH level increased, CNPs green synthesis increased.

### Role of incubation period on the green synthesis of CNPs

Figure 5C, E, and F depict the 3-D surface plots for the green synthesis of CNPs as a function of the incubation time and how it interacts with the other three variables: The graphs demonstrate that as the incubation period increase, the green synthesis of CNPs increased. Midway during the incubation period, most CNPs were produced in their green form (around 45 min.) Green synthesized CNPs production decreased with additional increases or decreases of incubation period. El-Naggar et al*.*^35^ found that after 57.53 min, the highest CNPs green synthesis using the leaves extract of *Pelargonium graveolens* was 9.73 mg/mL. According to de Oliveira et al*.*^108^, the incubation time is the least significant variable in determining the average size of the microspheres, as incubation should facilitate the fragmentation of the largest microspheres and increase the total number of particles. Also, the impact of stirring duration on particle size was examined, and the results showed that there were no substantial variations in the particle size distribution after 10, 20, or 45 min of agitation^88^.

### Role of chitosan concentration % on CNPs green synthesis

Figures 5A, B, and C show the three-dimensional response surface diagrams illustrating the impact of chitosan concentration percent on the green synthesis of CNPs and its influence on the subsequent variables of incubation period, initial pH, and temperature. As the chitosan concentration% attained its optimal level, the green synthesis of CNPs increased, as shown by the graphs. Maximum production of green synthesized CNPs (13.99 mg/mL) was generated at a high chitosan concentration % (about 1.6%). According to Ali et al*.*^107^, the size of nanoparticles increases as chitosan concentration rises. As chitosan concentration increases, the Zeta potential decreases, indicating fewer amino groups. As the surface Zeta potential decreases, the probability of particle aggregation likewise rises. Kamat et al.^109^ reported that chitosan concentrations of 0.8 mg/mL produced the highest nanoparticle production. The ideal starting chitosan concentration, as determined by Vaezifar et al*.*^110^, was 1.29 mg/mL. On the other hand, initial chitosan concentration significantly impacts nanoparticle size^111^*.* In El-Naggar et al.^35^ study, the optimum concentration of chitosan used for green synthesis of CNPs was 1.08%. At a chitosan concentration of 1%, the maximum yield of CNPs was achieved^14^.

### Role of temperature on the green synthesis of CNPs

Figures 5B, D, and F represent the 3-D surface plots for interactions between temperature and various variables on the green CNPs synthesis: initial pH, chitosan concentration, and incubation time. These graphs demonstrate that the green synthesis of chitosan nanoparticles increased as the optimal temperature was reached. At low temperatures, the highest concentration of CNPs produced by green synthesis (13.99 mg/mL) was observed. Handani et al*.*^111^ reported that the nanoparticles production decreases with increasing temperature, and the temperature variation does not significantly affect the initial size of nanoparticles produced or their growth during storage. Fan et al*.*^112^ discovered that when the temperature was increased from 10 °C to 60 °C, the particle size showed a clear tendency to decrease, whereas when the temperature exceeded 60 °C, the particle size decreased slightly. El-Naggar et al.^37^ mentioned that the optimum temperature used for the green synthesis of CNPs was 53.83°C. While the study of El-Naggar et al*.*^35^ found that the maximum yield for green synthesis of CNPs was 9.82 ± 3 mg/ml using temperature of 50.38 °C.

### The adequacy of the model

**Figure 6:**
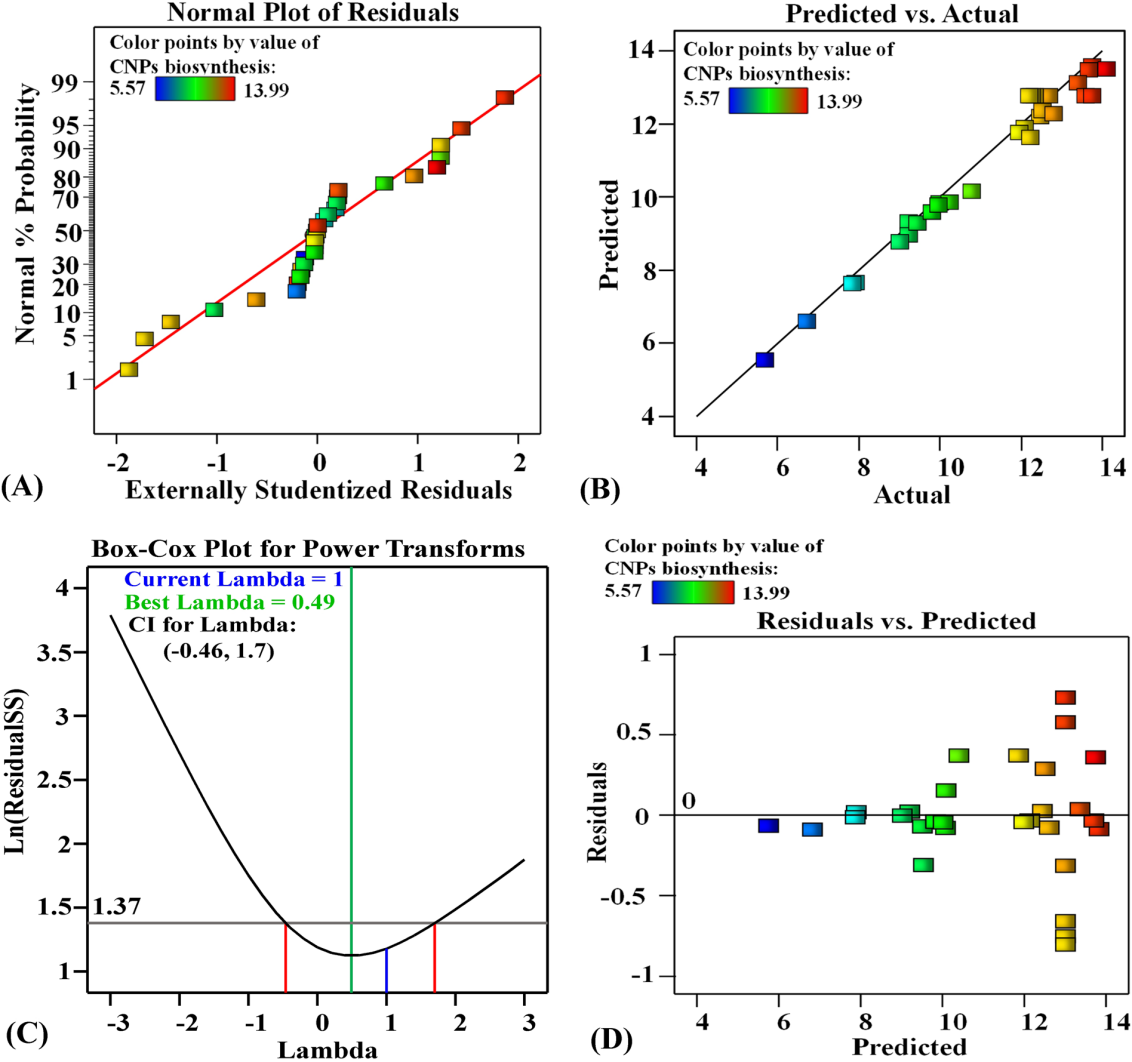
(**A**) Normal probability plot of internally studentized residuals, and (**B**) Plot of predicted versus actual of green synthesis of chitosan nanoparticles using *Cymbopogon citrates* extract, Box–Cox plot of model transformation (**C**), and (**D**) Plot of internally studentized residuals versus predicted values for green synthesis of chitosan nanoparticles using *Cymbopogon citrates* extract.

The normal probability plot (NPP), which displays the residual distribution, is a significant graphical tool for evaluating the model's adequacy^113^. The residuals are the variations between the predicted values by the theoretical model and the actual response values^114^. Figure 6A shows the NPP, which reveals that the residuals cluster relatively close to the diagonal straight prediction line of the green synthesized CNPs. Consequently, this demonstrates that the predicted values for the green synthesized CNPs are a good fit with the actual results, which demonstrates that the model is accurate^113^.

The predicted and actual values of the green synthesized chitosan nanoparticles are shown in Fig. 6B. The experimental results of the green synthesized CNPs show significant agreement with the theoretical values predicted by the model, validating its accuracy^115^. The Box–Cox plot for the green synthesized CNPs is presented in Fig. 6C. The green line represents the best lambda value (λ = 0.49), and the blue line represents the current transformation (value (λ = 1), while the two red lines represent the lowest and the highest values of 95% confidence intervals (-0.46 and 1.7, respectively). Figure 6C shows that the model is in the ideal zone because the current lambda blue line is located between the two red lines. Consequently, no data transformation is required^115,116^.

The predicted values of the green synthesized CNPs are displayed against the residual’s values (Fig. 6D). The residuals are distributed uniformly around the zero line, indicating the adequacy of the model. El-Naggar et al.^117,118^ reported that the residuals were randomly distributed around the horizontal zero reference line, indicating a good fit of the model.

### The desirability function (DF)

**Figure 7:**
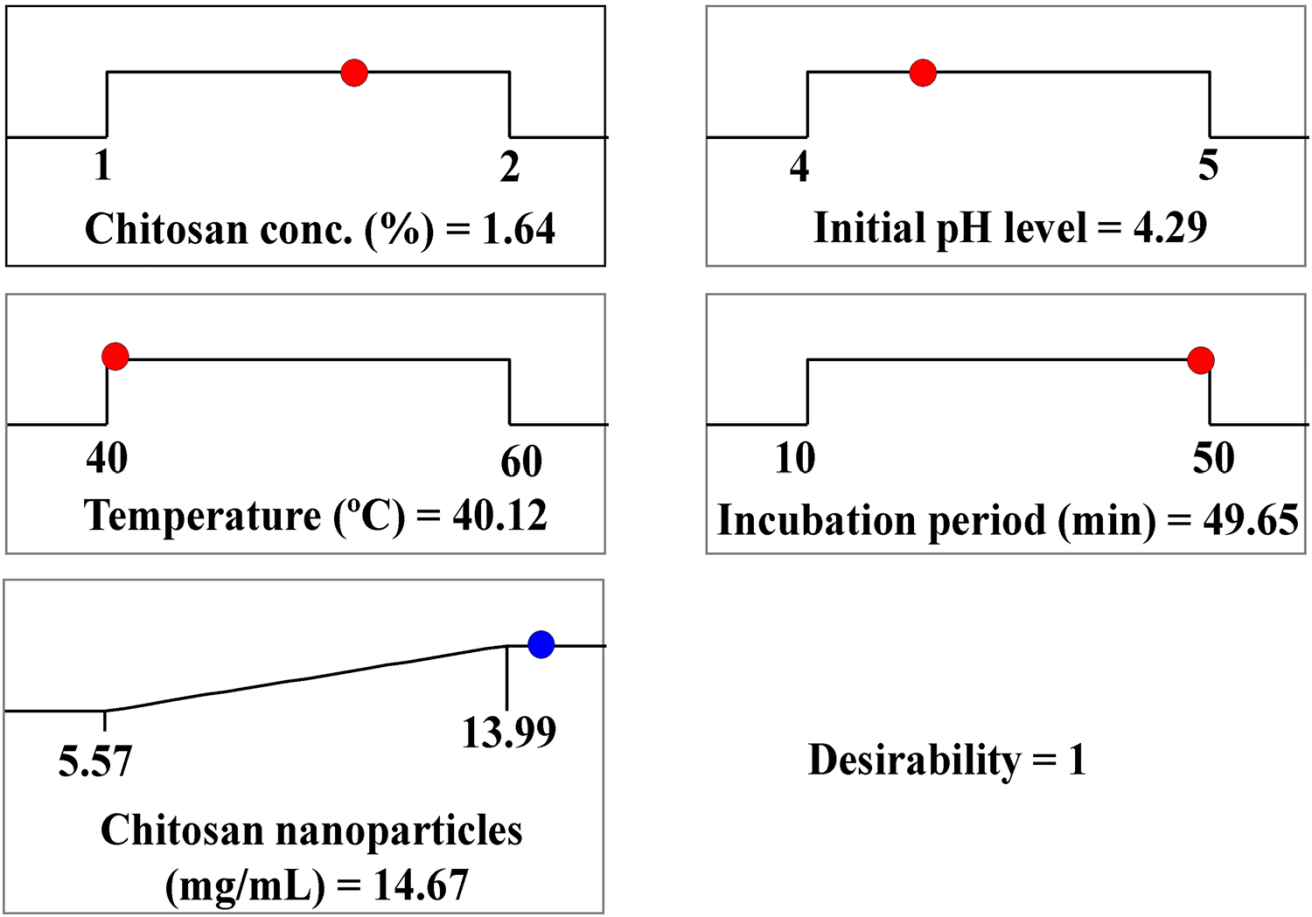
The desirability function (DF) and the optimum predicted values for the maximum green synthesis of chitosan nanoparticles using *Cymbopogon citrates* extract.

To determine the best conditions for maximizing the responses, the desirability function was used^119^. The DF values ranged from 0 (undesirable) to 1 (desirable)^120^. Figure 7 depicts the optimization plot, which shows the DF and the best predicted values for the investigated variables for the highest production of CNPs. In this research, the highest predicted value for green synthesized CNPs was 14.67 mg/mL using the predicted values for the investigated variables which were chitosan concentration (1.64%), initial pH level (4.29), temperature (40.12°C), and incubation time (49.65 min).

### Radial mycelial growth inhibition of *F. culmorum* by the green synthesized CNPs

**Figure 8:**
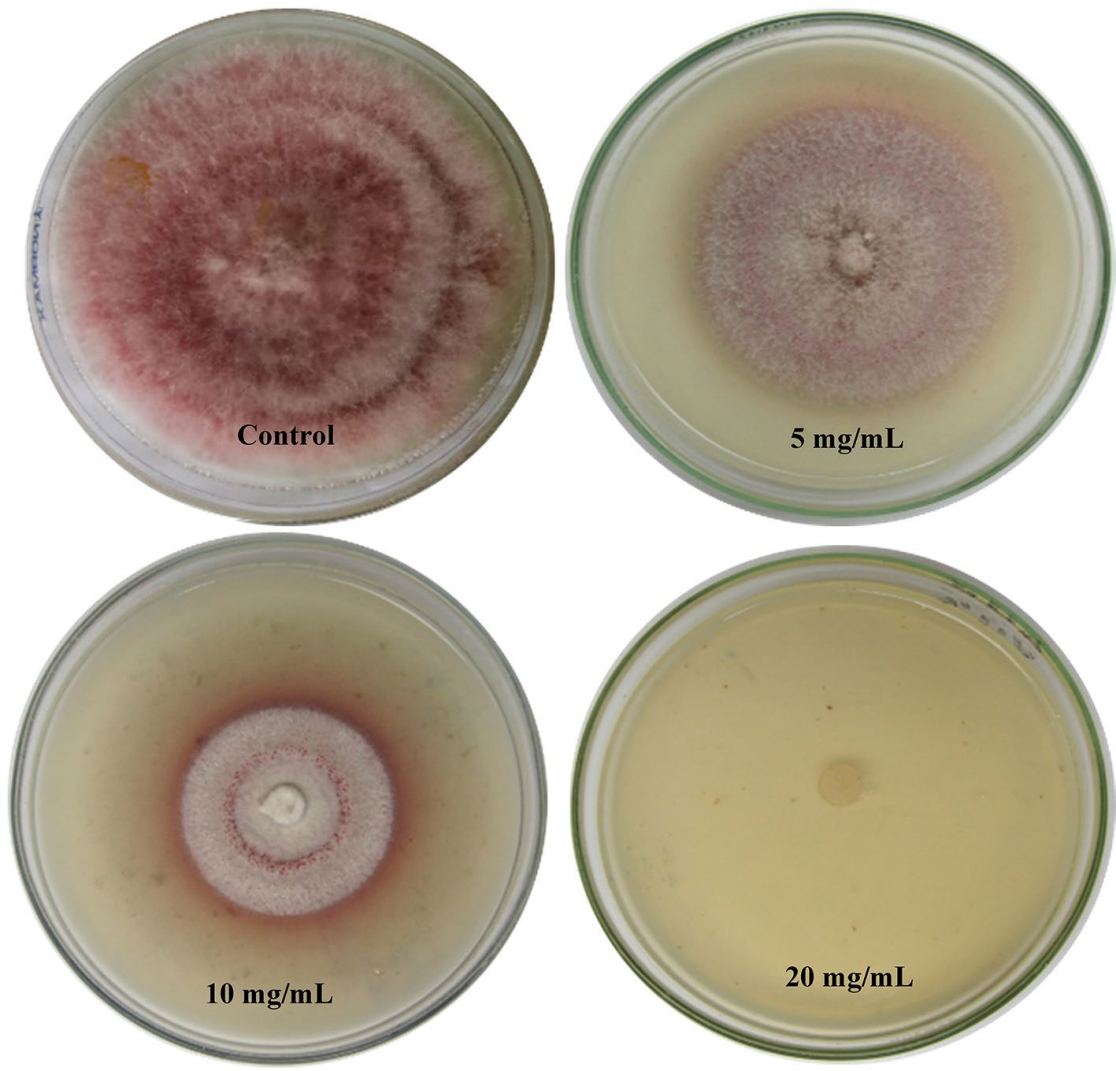
Antifungal activity of different concentrations of green synthesized chitosan nanoparticles produced using *Cympopogon citratus* extract against *Fusarium culmorum*.

Results in Fig. 8 show that all tested concentrations of the green synthesized CNPs had capability to inhibit linear growth of *F. culmorum*, the inhibition consistently increased with the CNPs concentration. The radial growth of *F. culmorum* was observed at 3 different concentrations (5 mg/mL, 10 mg/mL, and 20 mg/mL), after seven days of incubation. 20 mg/mL of CNPs caused a complete reduction (100%) of the mycelial growth, while the mycelial growth inhibition rate of 10 mg/mL concentration was 60% and 5 mg/mL was 31.11%. A number of chitosan concentrations (0.5, 1, 2, 4, and 8 mg/mL) inhibited the hyphal growth of *F. culmorum* f*.* sp. *cubense*, with a maximal inhibition of 76.4 percent at 8 mg/mL^121^.

The characteristics of chitosan, such as molecular weight, degree of deacetylation, and acetylation pattern can influence its antifungal activity^122^. The concentration and pH of chitosan water solutions also play a role in determining the final biological effect ^123^. CNPs have been shown to have enhanced antifungal activity compared to chitosan alone^58^. CNPs have a higher affinity to bind to fungal cells^124^. The antifungal activity of CNPs is attributed to several factors. Yien et al.^3^ demonstrated that chitosan nanoparticles exhibited higher antimicrobial activity due to their special characters of the nanoparticles such as small and compact particles as well as high surface charge. The response of fungi to chitosan and CNPs varies depending on the fungal species and their stages of development^122^. The mode of action of CNPs involves several mechanisms. Divya and Jisha^125^ reported that the positively charged CNPs interacts with the negatively charged phospholipids of the plasma membrane, changing cell permeability and causing cell death. This could be explained by the fact that the negatively charged plasma membrane is the main target site of polycation^3^. CNPs can disrupt the fungal cell membrane, inhibit ergosterol green synthesis, reduce protein and extracellular DNA content, and induce the production of reactive oxygen species (ROS)^126^. These mechanisms contribute to the antifungal and antibiofilm activities of chitosan nanoparticles against various fungal pathogens. As a primary cell death regulator, ROS play a critical role in cell death, and are involved in many of the vital psychological signaling pathways of filamentous fungi^127^. High ROS production in *Fusarium oxysporum* upon exposure to CNPs treatment could be the main reason for mycelium growth inhibition or death^128^. The enhancement of ROS production can imbalance the oxidant-antioxidant levels, increase the oxidative stress, and eventually releasing cytochrome C that leads to cell apoptosis^129^.

The antifungal activity of CNPs can be attributed to their larger surface area, which enables them to adsorb more tightly onto the surface of fungal cells and disrupt the membrane integrity^46^. Moreover, CNPs might be able to diffuse into fungal cell and hence disrupt the synthesis of DNA as well as RNA^130^, this could explain a better antifungal activity of CNPs compared to its free polymer or solution^3^. More importantly, exposure to CNPs led in the formation of an impermeable layer that blocked the channels on the cell surface, preventing the transportation of essential nutrients that are critical for the survival of microbial cells^131^.

The antifungal activity of the green synthesized CNPs showed strong inhibition against the severe phytopathogen (*Botrytis cinerea* SIB-1) with a wide host range and proved the ability of the CNPs to replace or minimize the extensive use of pesticides and also to be applied in various technological and medical fields^35^. On the other hand, Sathiyabama and Charles^132^ reported that CNPs synthesized from *Fusarium oxysporum* f.sp. *lycopersici*. using sodium tripolyphosphate exhibit antifungal activity under in vitro condition, protecting tomato plants from *F. oxysporum* f.sp. *lycopersici* infection. The foliar treatment to tomato plants attacked with *F. oxysporum* f. sp. *lycopersici* delayed the emergence of wilt disease symptoms, reduced the severity of the disease, and the treated plants also exhibited increased yield.

Chitosan was used as an eco-friendly preparation for controlling potato late blight, chitosan significantly inhibited the mycelial growth and spore germination of *Phytophthora infestans* in vitro and induce resistance in potato pieces and leaves to *Phytophthora infestans*, making it a potential way to reduce the use of chemical pesticides. chitosan mainly affected cell growth, and most of the Genes and Genomes pathways revolved in metabolic processes, cell membrane structure and function and ribosome biogenesis^133^. The possible mechanisms include inhibition of mycelial growth and sporogenesis, causing metabolic disorder of *Phytophthora infestans* and affecting multiple metabolic processes, destroying the structure and function of cell membrane. Moreover, chitosan can inhibit many physiological races of *Phytophthora infestans*, so it is a promising substitute for chemical pesticides^133^. CNPs enter the cell and link to the nucleic acids or proteins, alter the normal genetic information, causing abnormalities in the ribosome biogenesis, disrupts the cell function of the fungus, and finally resulting in the death of the cell^134^.

### In vitro antitumor activity test

A normal human lung fibroblast (WI38) cell line was used to compare the anticancer activity of the green produced CNPs to five cancer cell lines: colorectal carcinoma (HCT-116), epitheliod carcinoma (Hela), mammary gland (MCF-7), human prostate cancer (PC3), and hepatocellular carcinoma (HePG-2).

**Figure 9:**
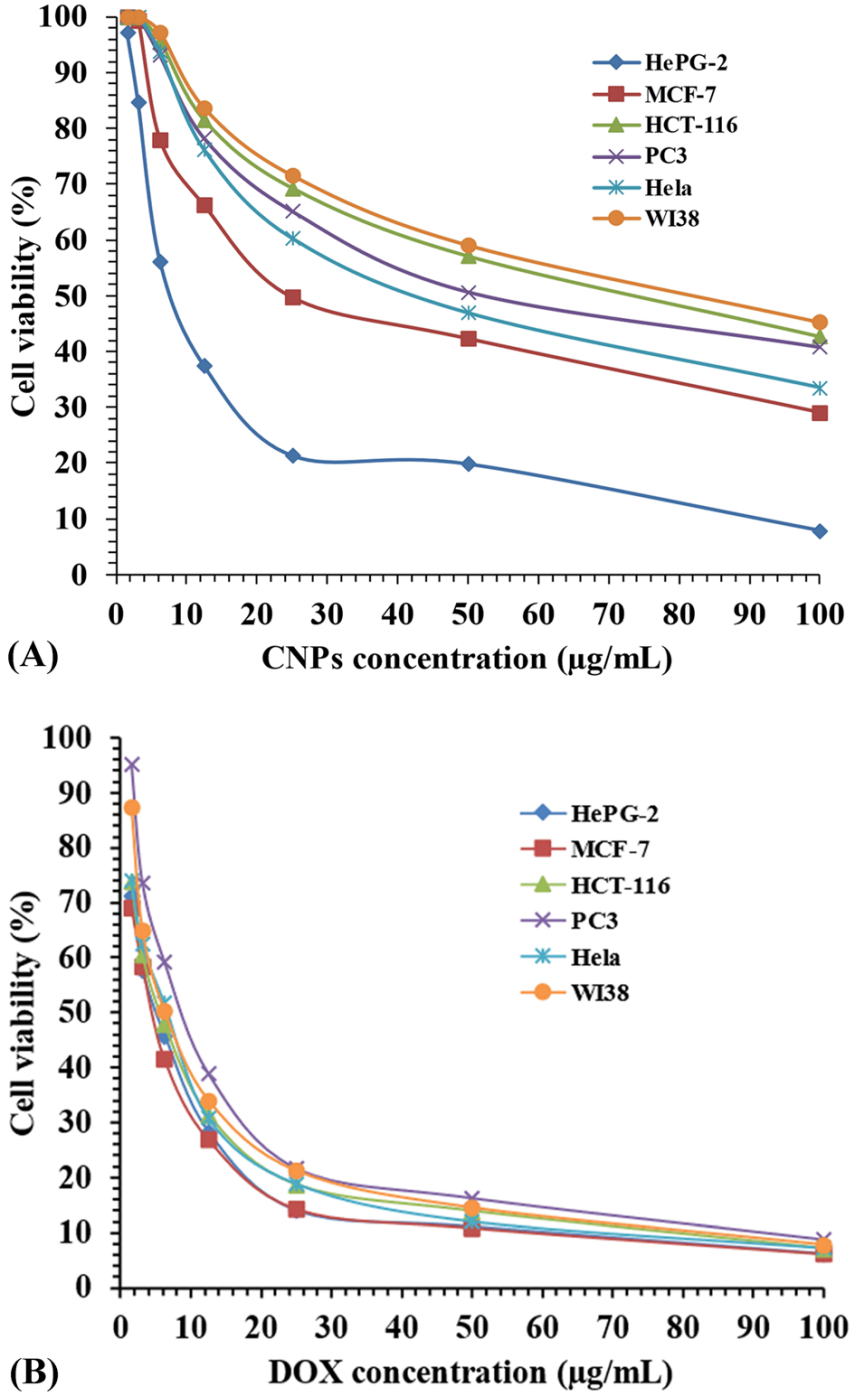
The anticancer effects (**A**) the green synthesized CNPs and (**B**) Doxorubicin on HePG-2, MCF-7, HCT-116, PC3, Hela and WI38 cells.

The viability of the cells decreases while increasing the concentration of green synthesized CNPs (Fig. 9). The lowest viability percentage (highest inhibition rate) of the all-cell lines was seen at a dose of 100 µg/mL. In the MCF-7 tumor cells, the maximum inhibition rate of green synthesized CNPs was approximately 70.9%, whereas the inhibition rate of Doxorubicin on the same cell line was 93.8%. The inhibition rates at 100 µg/mL concentration of CNPs were 68.8, 57.3, 59.2, and 66.5% for the tumor cells HePG-2, HCT-116, PC-3, and Hela; respectively, while the inhibition rate of Doxorubicin on the same cell lines was 93.7, 92.9, 91.2, and 92.7%; respectively. The normal cell line (WI38) showed inhibition rate of 54.7% for green synthesized CNPs, and 92.2% for Doxorubicin. The IC_50_ dose of the green synthesized CNPs on the examined cells HePG-2, MCF-7, HCT-116, PC-3, Hela and WI-38 were 36.25 ± 2.3, 31.21 ± 2.2, 67.45 ± 3.5, 56.30 ± 3.3, 44.62 ± 2.6 and 74.90 ± 3.8; respectively.

According to Rajivgandhi et al.^135^, when chitosan nanoparticle-loaded essential oils were released into the cells, the nucleus was directly affected and the genes that cause a high number of apoptosis formation were promoted. When cancer cells exposed to chitosan nanoparticles, apoptosis could continue, potentially leading to the death of a large number of cancer cells. Wang et al.^136^ also mentioned that the anti-cancer activities of chitosan nanoparticles were found to be concentration-dependent and effective against a variety of cancer cells. Chitosan’s antiangiogenic mechanism explains it’s antitumor activity. Through this process pathological conditions interfere with the reciprocal regulation of proangiogenic and antiangiogenic factors^137^. Hosseinzadeh et al.^138^ informed that HT-29 colon carcinoma cell line viability is inhibited by chitosan nanoparticles. Xu et al.^139^ showed that human hepatocellular carcinoma may be inhibited by chitosan nanoparticles (CNPs) via a mechanism of CNP-mediated regulation of tumor angiogenesis that was attributed to a reduction in vascular endothelial growth factor receptor 2 levels. Chitosan nanoparticles have been shown to suppress the proliferation of human hepatoma BEL7402 cells in vitro by necrosis caused by surface charge neutralization, penetration across the cell membrane, reduction in MMP, and induction of lipid peroxidation^140^. It has been documented that chitosan nanoparticles exhibit a propensity to accumulate preferentially in tumor cells due to their improved permeation and retention properties. Moreover, these nanoparticles reduce multidrug resistance induced by *p*-glycoprotein^141^.

Overall, the promising in vitro anticancer activities of the green synthesized CNPs by *Cymbopogon citrates* leaves extract against HePG-2, HCT-116, PC-3, and Hela cell lines in the current study, making them promising candidates for further validation studies in vivo in the next manuscript, as more extensive research in this area is required.

## Data Availability

All data generated or analyzed during this study are included in this article.
